# miR-140-5p could suppress tumor proliferation and progression by targeting TGFBRI/SMAD2/3 and IGF-1R/AKT signaling pathways in Wilms’ tumor

**DOI:** 10.1186/s12885-019-5609-1

**Published:** 2019-04-29

**Authors:** Zhuo Liu, Feng He, Shengrong OuYang, Yuanyuan Li, Feifei Ma, Huibo Chang, Dingding Cao, Jianxin Wu

**Affiliations:** 1 Department of Biochemistry & Immunology, Capital Institute of Pediatrics, NO. 2, Yabao Road, Chaoyang District, Beijing, 100020 China; 2 Graduate School of Peking Union Medical College, NO. 9, Dongdansantiao, Dongcheng District, Beijing, 100730 China

**Keywords:** Wilms’ tumor, miRNA-140-5p, IGF1R, TGFBRI, cell signaling

## Abstract

**Background:**

Wilms’ tumor is also called nephroblastoma and is the most common pediatric renal cancer. Several genetic and epigenetic factors have been found to account for the development of Wilms’ tumor. MiRNAs play important roles in this tumorigenic process. In the present study, we aimed to investigate the role of miR-140-5p in nephroblastoma by identifying its targets, as well as its underlying molecular mechanism of action.

**Methods:**

The miRNA expression profile of nephroblastoma samples was investigated and the targets of miR-140-5p were predicted and validated using the miRNA luciferase reporter method. Moreover, the roles of miR-140-5p in regulating nephroblastoma cell proliferation, migration and cell cycle were analyzed by the CCK8, migration and flow cytometry assays, respectively. The downstream protein of the direct target of miR-140-5p was also identified.

**Results:**

miR-140-5p was downregulated in Wilms’ tumor tissues, whereas in the nephroblastoma cell lines G401 and WT-CLS1 that exhibited high levels of miRNA-140-5p, inhibition of cellular proliferation and metastasis were noted as well as cell cycle arrest at the G1/S phase. TGFBRI and IGF1R were identified as direct target genes for miRNA-140-5p. In addition, SMAD2/3 and p-AKT were regulated by TGFBRI and IGF1R separately and participated in the miRNA-140-5p regulatory network. Ectopic expression of TGFBR1 and IGF-1R could abrogate the inhibitory effect of miR-140-5p.

**Conclusion:**

We demonstrated that miRNA-140-5p participates in the progression of Wilms’ tumor by targeting the TGFBRI/SMAD2/3 and the IGF-1R/AKT signaling pathways.

## Background

Nephroblastoma is one of the most common solid tumours in children, with an annual incidence rate of 1 case per 100,000 children. This disease comprises 8–10% of all neoplasms in that group [1, 2]. The peak incidence occurs in children, between 1 to 5 years of age [1, 2]. Although the pathogenesis of nephroblastoma remains undiscovered, increasing evidence has suggested that multiple signalling pathways, such as microRNAs, and epigenetic mechanisms play pivotal roles in its progression.

MicroRNAs (miRNAs) are endogenously produced, small (17~25-nucleotides long), non-coding single-stranded RNAs that play important roles in the regulation of crucial biological processes including cell apoptosis, metabolism, inflammation and tumorigenesis, primarily by inhibiting gene expression [3]. MiRNAs regulate the expression of mRNA molecules by binding to the complementary sequence in 3′-untraslated regions (3′UTRs) or the open reading frames of target genes [4, 5]. It has been studied that altered expression of miRNAs contributes to the initiation, invasion and metastasis of various types of cancer [6, 7]. miRNA 140–5p (miR-140-5p) has been shown to participate in various tumor processes. Yang et al. demonstrated that miR-140-5p could suppress tumor progression by targeting TGFBRI and FGF9 in hepatocellular carcinoma [8]. Yunfeng et al. demonstrated that miR-140-5p suppressed tumor growth and metastasis of non-small cell lung cancer by targeting IGF1R [9]. However, the function of miR-140-5p in the progression of nephroblastoma has not been explored.

TGFBRI and IGF1R can participate in the regulation of cell proliferation, differentiation, invasion and migration of cancer cells [10]. IGF1R is a tyrosine kinase receptor, which binds to IGF1 and IGF2. Following binding, the receptor auto-phosphorylates on the corresponding tyrosine residues [11, 12]. TGFBRI is a serine/threonine kinase receptor that is a member of the TGF-β signaling pathway, and exhibits metastatic properties by invading surrounding cells [13]. Previous reports have indicated that the TGF-β and IGF signaling pathways are associated with the development of nephroblastoma [14, 15]. However, the roles of TGFBRI and IGF1R in the progress of nephroblastoma require further investigation.

The purpose of the present study was to detect the potential role of miR-140-5p in the development of nephroblastoma, and the regulatory mechanism of this interaction.

## Methods

### Cell culture and clinical tissue specimens

The human nephroblastoma cell lines G401 and WT-CLS1 were matained in our labs and both were cultured in McCoy’s 5A medium containing 10% fetal bovine serum, 100 IU/ml penicillin and 100 mg/ml streptomycin. HEK-293 T cell line was maintained in DMEM supplemented with 10% fetal bovine serum, 100 IU/ml penicillin and 100 mg/ml streptomycin. All cells were cultured in a humidified atmosphere containing 5% CO_2_ at 37 °C.

Nephroblastoma tissue and adjacent non-cancerous tissue (ANT) samples were obtained from nephroblastoma children undergoing tumor resection at the Department of Surgery in the Affiliated Children’s Hospital of the Capital Institute of Pediatrics. The patients were recruited from September 2010 to April 2013. The tissue samples were immediately frozen in liquid nitrogen, and stored at − 80 °C until further analysis [16]. All the patients did not receive chemotherapy before surgical resection. The study was approved by the Ethics Committee for clinical research of the Capital Institute of Pediatrics.

### miRNA Array

miRNA expression profile of tumor tissue samples and ANT were detected by miRCURY LNA miRNA chips 16.0 (Exiqon, Vedbaek, Denmark). The assay was conducted following the protocol of the manufacturer. Image analysis was processed by using the image analysis software Genepix Pro 6.0 (Axon Instruments) as described [17].

### RNA purification and quantitative Real-Time PCR (qRT-PCR)

Total RNA was isolated from cells and tissue specimens with TRIzol reagent (Invitrogen, USA) as performed by the manufacturer’s instructions [18]. Quantitative real-time RT-PCR analysis was used to determine the level of mature miR-140-5p. One microgram of total RNA sample was reverse-transcribed to first-strand cDNA with the PrimeScript™ RT reagent kit as described by the manufacturer (Takara, Dalian, China). Real-time -PCR was conducted in triplicate, using SYBR Premix Ex Taq™ (Takara). The amplification was carried out on a 7900HT system with the SYBR Premix Ex Taq™ (Takara). The primer sequences used for quantitative real-time -PCR analyses of miRNA-140-5p were as follows: forward, 5’CTCAACTGGTGTCGTGGAGTCGGCAATTCAGTTGAGCTACCA3’ and reverse, 5′ ACACTCCAGCTGGGCAGTGGTTTTACCCTATG3’. The primer sequences used for small nuclear RNA (U6) were as following: forward, 5′-CAAATTCGTGAAGCGTTCCATAT-3′ and reverse 5′-GTGCAGGGTCCGAGGT-3′. The 2-∆∆Ct method was used to examine the relative expression levels of miRNAs. Specific siRNAs used to silence IGF-1R and TGFBRI gene were obtained from CST (CST, USA).

### Lentiviral packaging of miR-140-5p

Oligonucleotides of miRNA-140-5p were synthesized, based on the sequence of human miRNA-140-5p (5′- cagugguuuuacccuaugguag − 3′, MIMAT0000431) from the miRBase database. The oligonucleotides were introduced into a pGCSIL-GFP plasmid (GeneChem Co. Ltd. Shanghai, China). pHelper 1.0 and pHelper 2.0 vector used for lentiviral construction were also obtained from Shanghai GeneChem Co. Ltd.. The generation of lentiviruses and the evaluation of the viral titration were conducted as described previously [19]. Lentiviruses carrying miR-140-5p and/or negative control sequences were packaged following the instructions of the manufacturer (Shanghai Genechem Co., Ltd., Shanghai, China).

### Luciferase Reporter Assay

The plasmids used for firefly luciferase reporter assay were packaged by Genechem (Shanghai Genechem, China). And the plasmids designated IGF1R-WT, TGFBRI-WT (wild-type of miR-140-5p, targeting to IGF1R 3′-UTR and TGFBRI 3′-UTR), IGF1R-MU and TGFBRI-MU (mutated miR-140-5p, targeting to IGF1R 3′-UTR and TGFBRI 3′-UTR) were used. The mimic and the mimic negative control of miR-140-5p were purchased from Ribobio (Guangzhou RiboBio, Guangzhou, China). HEK293 cells were cultured in complete medium for 24 h before transfection. 0.05 μg firefly luciferase reporter, 0.05 μg IGF1R/TGFBRI plasmid, and 0.01 μg Renilla luciferase control vector were co-transfected into HEK293 cells by lipofectamine 2000 (Invitrogen, Carlsbad, CA) following the manufacturer’s instructions. At 48 h post-transfection, luciferase activity was detected by using the Dual-Glo luciferase reporter system (Promega Corp., Madison, WI, USA) in accordance with the protocols of manufacturer [20]. The relative luciferase activity value was achieved against the renilla control.

### miRNA pull down assay

Biotin-coupled miRNA and mRNA pull-down assays were carried out as described in detail elsewhere [21]. Briefly, Biotinylated miRNA-140-5 probe were generated: (cagugguuuuacccuaugguag-biotin and control biotinylated probe: cuaccauaggguaaaaccacug-biotin, Generay,Shanghai,China). Biotin-coupled RNA probe and G401 cell total RNA were incubated in RNA buffer without RNAase at 4 °C overnight. Then the biotin-coupled RNA complexes were pulled down by using M-280 Streptavidin Dynabeads (Invitrogen, Carlsbad, CA). TGFBR I and IGF1R abundance in the bound fractions was calculated by RT-PCR analysis.

### Cell proliferation assay

Cell proliferation viability was measured using the cell counting kit-8 assay kit (Dojindo Laboratories, Japan) as described. Cells were seeded in 96-well plates, and incubated for 48, 72 and 96 h at 37 °C after transfection. The CCK-8 solution was added to each well at the end of incubation, and then cells were further cultured at 37 °C for an additional 1.5 h. Subsequently, the absorbance (450 nm) value of each well was estimated by a microplate reader.

### Cell cycle analysis

Flow cytometry was used for cell cycle analysis with a Propidium Iodide (PI) cell cycle detection kit following the manufacturer’s protocols (Transgen Biotech, Beijing, China).

### Migration assay

The Ibidi cell migration (gap closure/cell-exclusion zonemigration assays) technology (Ibidi, Martinsried, Germany) was used for the migratory assay. Briefly, G401 or WT-CLS1 cells were cultured, and then miRNA was transfected into the cells. Subsequently, the cells were collected with trypsin and washed with PBS, and then resuspended in DMEM containing 10% FBS (~ 7.0 × 10^5^ cells/ml). Next, 70 μl cell suspension was added to the wells of the culture-insert on a 35-mm dish, cultured in a incubator with 5% CO_2_ atmosphere at 37 °C. At 24 h after incubation, the culture-insert was removed using sterilized tweezers. Then cell migration was observed over time. At 12 h after culture, the increase of the area was calculated using ImageJ.

### Western Blot Analysis

The cells or nephroblastoma tissue were homogenized and lysed in ice-cold RIPA lysis buffer (Kangwei, Beijing, China) including protease and phosphatase inhibitors. The cell debris was removed by centrifuging at 14,000×g for 20 min at 4 °C. The protein samples were prepared and separated by 12% SDS-PAGE, and then electrophoretically transferred to polyvinylidene difluoride (PVDF) membranes (Millipore, Bedford, MA) [16]. Subsequently, the membranes were incubated for 12 h at 4 °C with primary antibodies, including anti-IGF1R, anti-p-AKT, anti-Smad2/3 anti-GAPDH (CST, USA) and anti-TGFBRI (R&D, USA) antibodies following by incubation with secondary antibodies. The binding of all antibodies was visualized using enhanced chemiluminescence (ECL) western blotting detection system (Amersham Life Science, Piscataway, NJ, USA) following the manufacturer’s protocol. GAPDH was utilized as a loading control.

### Statistical Analysis

The experiments were performed in triplicate. The data were analyzed by GraphPad Prism 5 (La Jolla, CA, USA). Statistical analysis was performed by using SPSS Statistical Package (v. 13.0, SPSS Inc., Chicago, IL). The Mann-Whitney U test was used for the analysis of the expression data of miR-140-5p. The Student’s t-test was utilized for unpaired sample comparison. Statistical significance levels were set at *P* < 0.05.

## Results

### MicroRNA-140-5p is downregulated in nephroblastoma tissues and is associated with clinical outcome

**Fig. 1:**
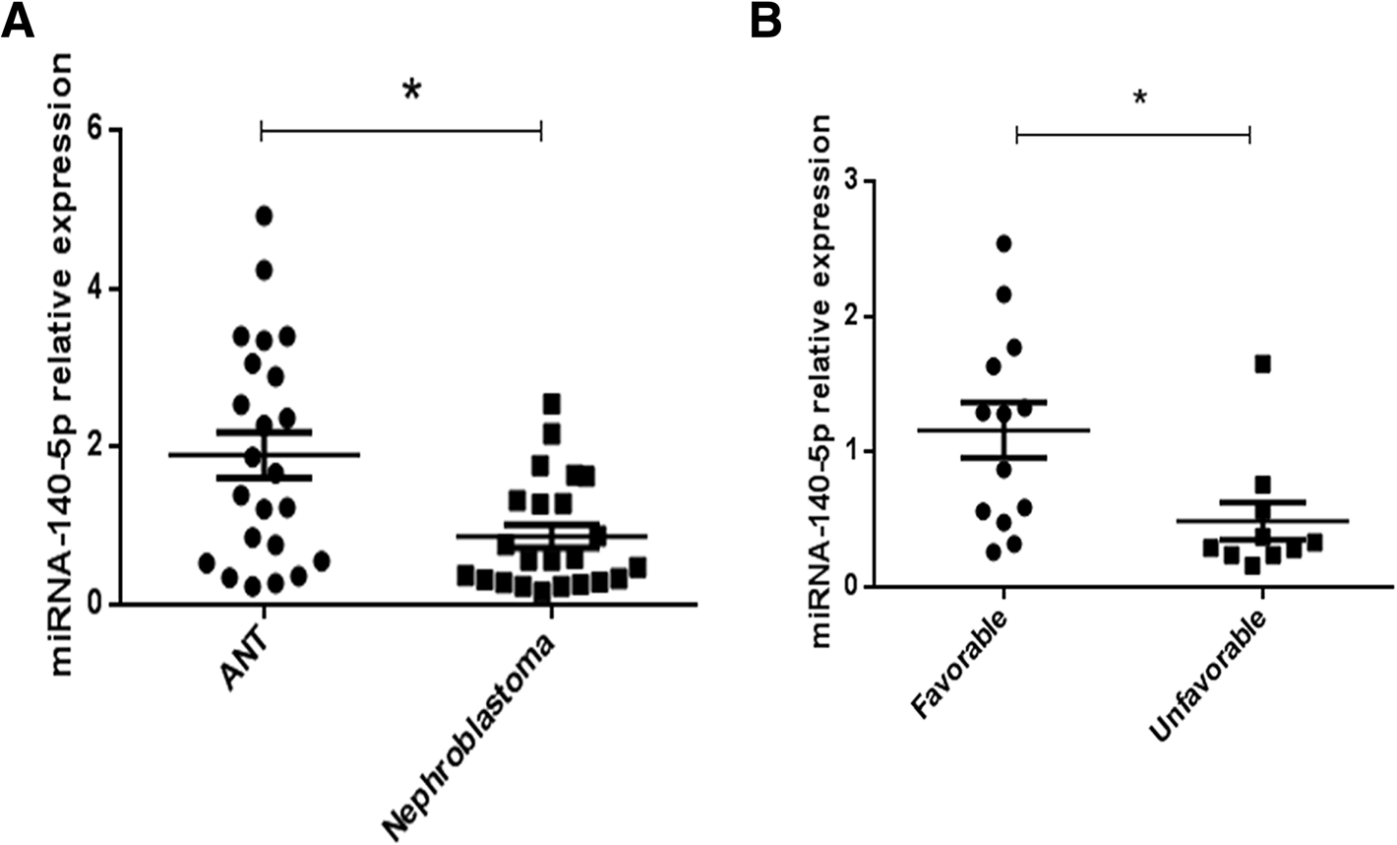
Downregulated miRNA-140-5p expression correlated with poor clinical outcomes of nephroblastoma. Relationship between miRNA-140-5p levels and clinical character of Wilms’ tumor were detected. (**a**) The expression levels of miRNA-140-5p were analyzed in nephroblastoma and corresponding normal tissues by real-time PCR. U6 served as the control for RNA loading. (**b**) The clinicopathological characteristics of the nephroblastoma patients were analyzed according to the expression levels of miRNA-140-5p.^*^P < 0.05

miRCURY LNA microRNA chips from Exiqon was used to analyze three cases of nephroblastoma and adjacent normal tissue (ANT) obtained by surgical removal. miRNA microarray analysis revealed that miR-140-5p was significantly downregulated in nephroblastoma tissues compared with the corresponding levels of expression in ANT. qRT-PCR was performed in 23 cases of nephroblastoma tissues and 23 ANTs. qRT-PCR analysis revealed that the expression of miRNA-140-5p was significantly decreased in nephroblastoma tissues compared with that of the control group (Fig. 1a). To assess the feasibility of miR-140-5p expression in nephroblastoma prognosis, we analyzed the correlation between the clinicopathological characteristics of Wilms’ tumor patients and the miRNA-140-5p levels. The data suggested that patients with higher tumor stage and unfavorable histology exhibited lower levels of miRNA-140-5p (Fig. 1b). The results indicated that miRNA-140-5p may participate in Wilms’ tumor progression.

### Overexpression of microRNA-140-5p inhibits proliferation and metastasis of nephroblastoma cell lines

**Fig. 2:**
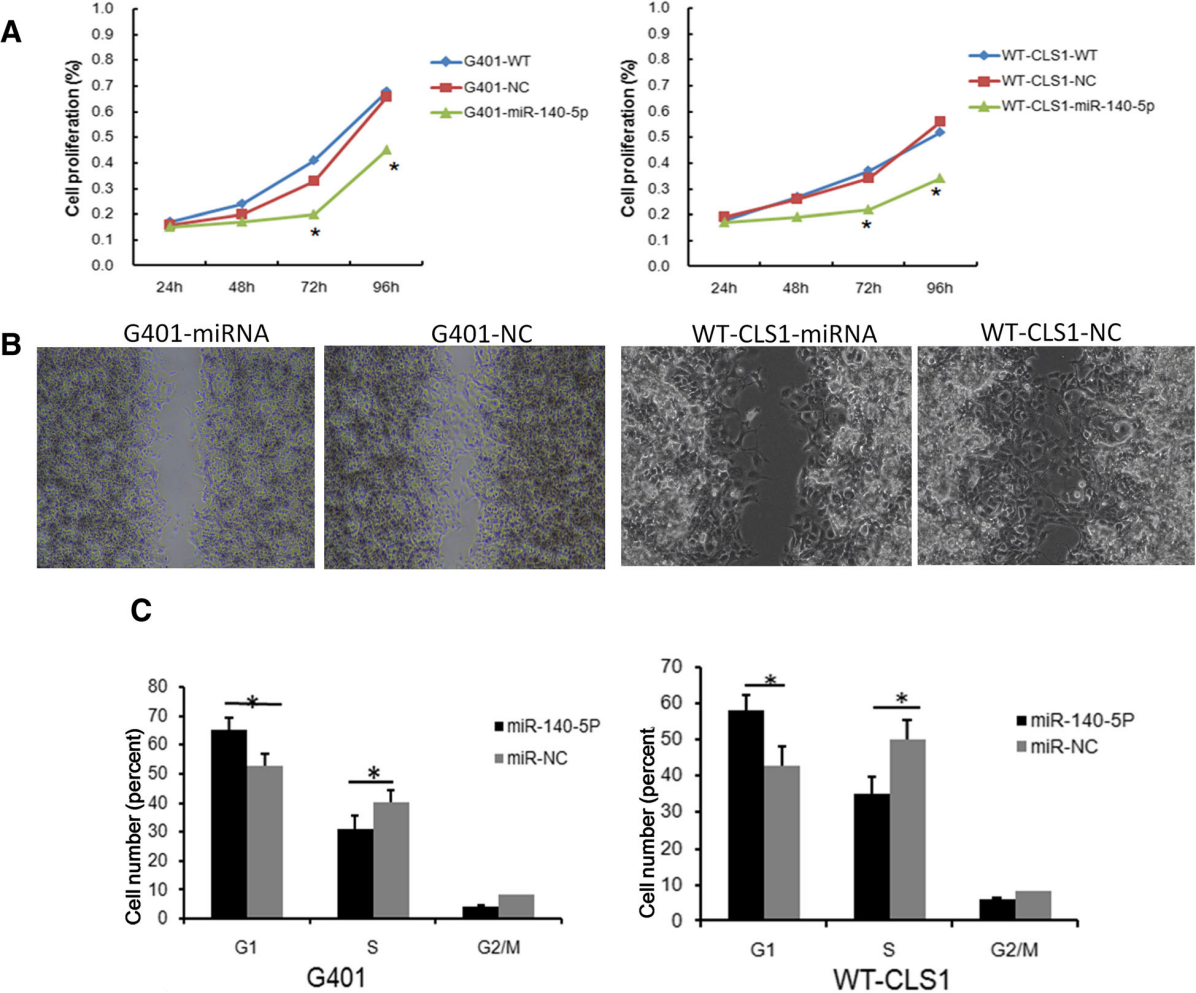
miRNA-140-5p suppresses nephroblastoma cells growth in vitro. Lentivirus containing miRNA-140-5p were transduced into G401 and WT-CLS1 cells, cell proliferation, migratory ability and cell cycle were tested. **a** The proliferation of G401 and WT-CLS1 cells was downregulated in the miRNA-140-5p group by the CCK8 assay. **b** The migratory ability of G401 and WT-CLS1 cells were inhibited in the miRNA-140-5p group, representative photographs of cell migration assay were shown. **c** G401 and WT-CLS1 cells were arrested in the G1/S phase of the cell cycle in the miRNA-140-5p group. ^*^P < 0.05

To further assess the impact of miR-140-5p on tumor growth and metastasis, we performed cell proliferation and migration assays. Two nephroblastoma cell lines (G401 and WT-CLS1) were infected with lentivirus containing miR-140-5p sequence, or control lentivirus containing non-specific sequences at an MOI = 10. MiRNA-140-5p expression levels were higher in the miR-140-5p transfection group than those in the control group (data not shown). Cell proliferation and migration assays were performed. The proliferation assay indicated that lentiviral-induced ectopic miR-140-5p resulted in a significant decrease in cell proliferation in both G401 and WT-CLS1 cells (Fig. 2a). The migration assay indicated that overexpression of miR-140-5p significantly suppressed the migratory abilities of the nephroblastoma cell lines (Fig. 2b). We subsequently performed cell cycle analysis and revealed that overexpression of miR-140-5p decreased the percentage of cells in the S phase in G401 and WT-CLS1 cells, while it concomitantly increased the percentage of cells in the G1 phase (*P* < 0.05) (Fig. 2c).

### TGFBR I and IGF-1R are candidate targets for miRNA-140-5p

**Fig. 3:**
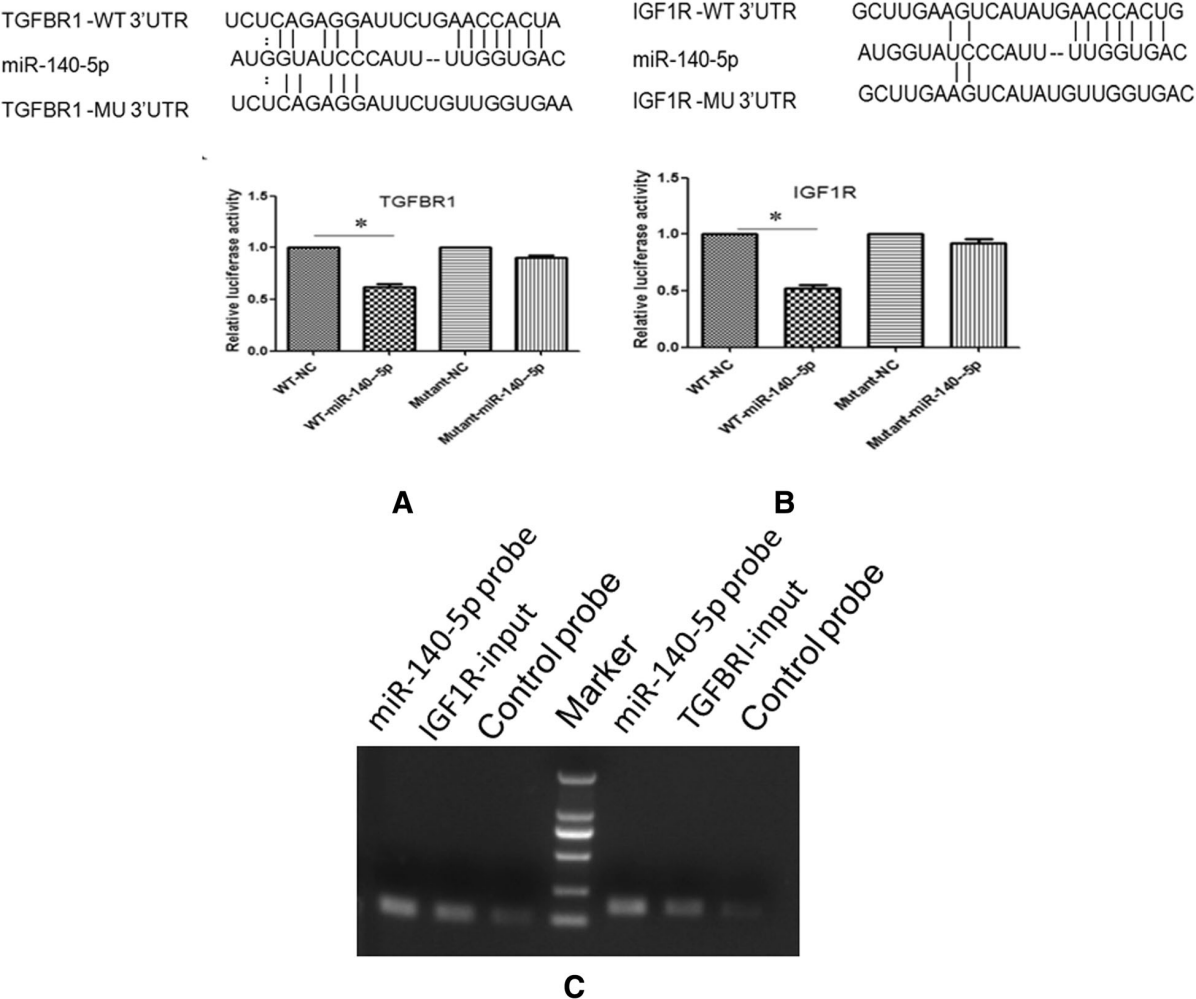
miRNA-140-5p could target TGFBRI and IGF1R. Validation of TGFBRI and IGF1R as targets of miRNA-140-5p. The sequence of potential binding site of miRNA-140-5p in the 3’UTR of TGFBRI and IGF1R mRNA (**a**) and (**b**). Validation of the miRNA-140-5p binding target using luciferase reporter assays. HEK-293 T cells were transfected with an miRNA-140-5p mimic or a control oligonucleotide (NC) and a dual luciferase reporter containing a wild-type promoter or a mutant promoter of TGFBRI and IGF1R. The luciferase activities in the cells with the mutant promoter/NC were significantly higher than those with the miRNA-140-5p mimic-wild-type reporter (**a** and **b**). TGFBRI and IGF1R in G401 cell lysis was pulled down and enriched with a miRNA-140-5p-specific probe and then detected using RT-PCR, the amount of IGF1R and TGFBRI were higher in the miR-140-5p probe group than control group(**c**)

To explore the underlying mechanism of miR-140-5p in nephroblastoma, we performed a bioinformatics search for candidate targets of miR-140-5p in genes that were involved in nephroblastoma pathogenesis using the TargetScan (http://www.targetscan.org), PicTar (http://www.pictar.org/) and miRnada (http://miranda.org.uk/) databases. The analysis indicated that miR-140-5p possibly regulated the IGF1R and TGFBRI genes since their 3′-UTR included the binding sites for the seed region of miR-140-5p (Fig. 3a and b).

To verify this speculation, HEK293 cells were transfected with miR-140-5p, mimic control, the 3′-UTR wild type of the IGF1R/TGFBRI gene (WT-3′-UTR), and its mutant form (MU-3′-UTR). The results demonstrated that miR-140-5p mimic specifically reduced the activity of a reporter containing the IGF1R/TGFBRI-3′-UTR-WT(*P* < 0.05), while no effect was apparent in the miR-NC or IGF1R/TGFBRI-3′-UTR-MU groups, implying the inhibitory effect of miR-140-5p on the IGF1R and TGFBRI genes (Fig. 3a and b).

**Fig. 4:**
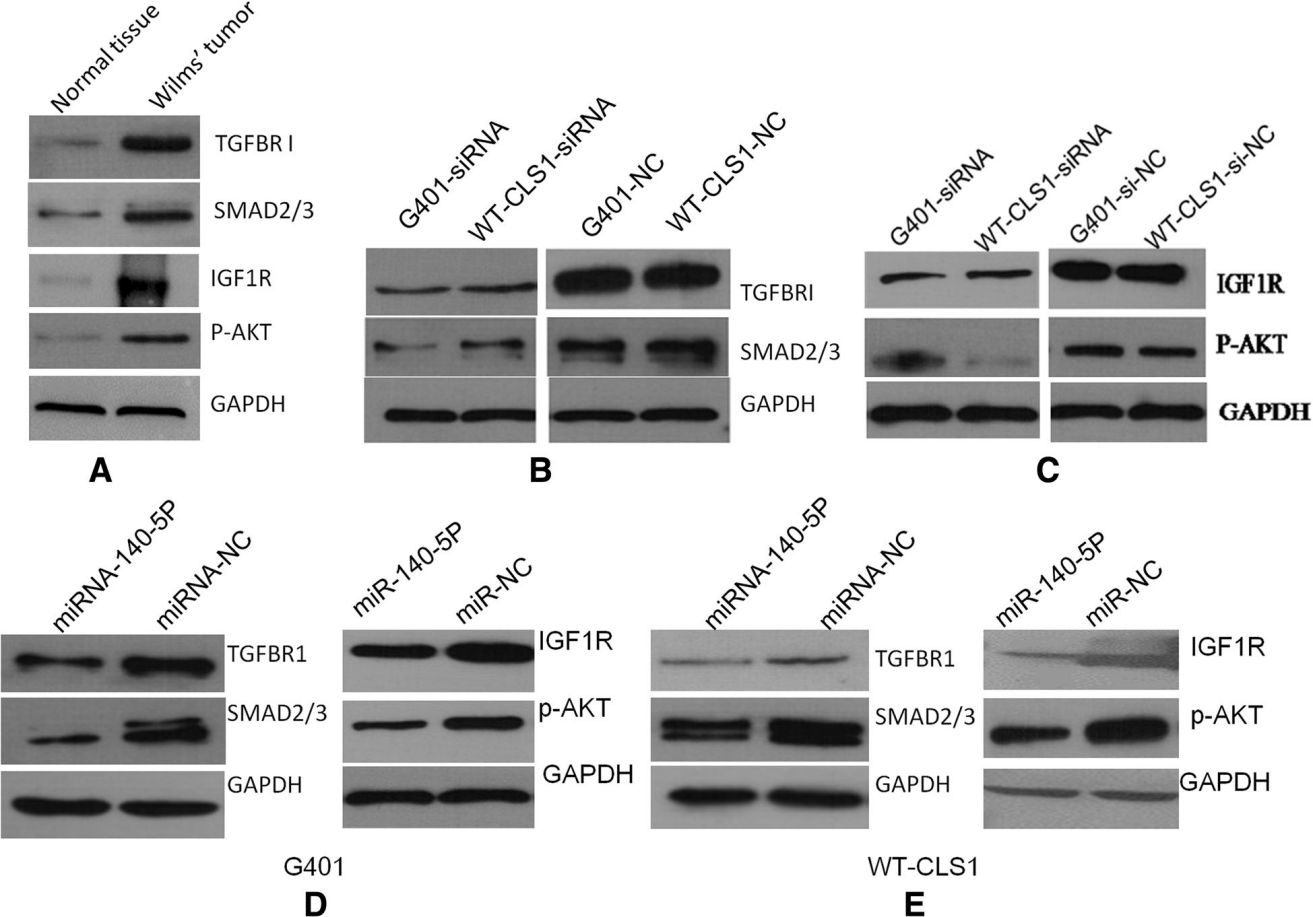
MicroRNA-140-5p suppresses IGF-1R and TGFBR I signaling pathways. Expression of the main proteins in IGF-1R and TGFBR I signaling pathways were detected in tumor tissues, siRNA transfected cells and microRNA-140-5p overexpressed cells. TGFBRI/SMAD2/3 and IGF1R/P-AKT expression were detected in the nephroblastoma tissues and adjacent normal tissues, shown were representative results from 3 experiments (**a**). SMAD2/3 expression was downregulated by inhibition of TGFBRI (**b**), whereas AKT activation was downregulated by inhibition of IGF1R (**c**) in G401 and WT-CLS1 cells. MicroRNA-140-5p could suppress TGFBRI/SMAD2/3 and IGF1R/P-AKT expression in G401 (**d**) and WT-CLS1 cells (**e**)

In order to investigate whether miR-140-5p could bind to IGF1R/TGFBRI, we used a miR-140-5p-specific probe to pull down its associated genes in G401 cells. RT-PCR results showed that IGF1R and TGFBRI were greatly enriched in the miR-140-5p precipitation complex (Fig. 3c). Thus, IGF1R and TGFBRI were identified as direct target genes of miR-140-5p. We also found that IGF1R and TGFBRI expression in nephroblastoma tissue were higher than that of adjacent normal tissues (Fig. 4a).

### MiRNA-140-5p represses IGF-1R and TGFBR I signaling to suppress nephroblastoma growth and metastasis

Based on the finding that miRNA-140-5p could suppress cell proliferative and metastatic activities, we further evaluated the function of the target genes IGF-1R and TGFBR1 in G401 and WT-CLS1 cells by specific siRNA-silencing. IGF-1R-siRNA and TGFBR1-siRNA could decrease cell proliferation and metastasis **(data not shown)**. The expression levels of SMAD2/3 and p-AKT were further evaluated in siRNA-transfected cells. SMAD2/3 is a downstream mediator of TGFBR1 and the activation of AKT is considered the main downstream signal of IGF-1R, both SMAD2/3 and p-AKT participate in the process of cancer progression by promoting cell growth, anti-apoptotic effects, and cell invasion [22, 23]. To verify importance of both proteins in Wilms’ tumor, we detected expression of SMAD2/3 and p-AKT and found that both were highly expressed in the tumor tissues compared with adjacent normal tissue (Fig. 4a). We also found that SMAD2/3 and p-AKT were downregulated in TGFBR1- knockdown cells and IGF-1R-knockdown cells, respectively (Fig. 4b and c). Furthermore, SMAD2/3 and p-AKT levels were both downregulated in miR-140-5p-overexpressing G401 and WT-CLS1 cells (Fig. 4d and e).

**Fig. 5:**
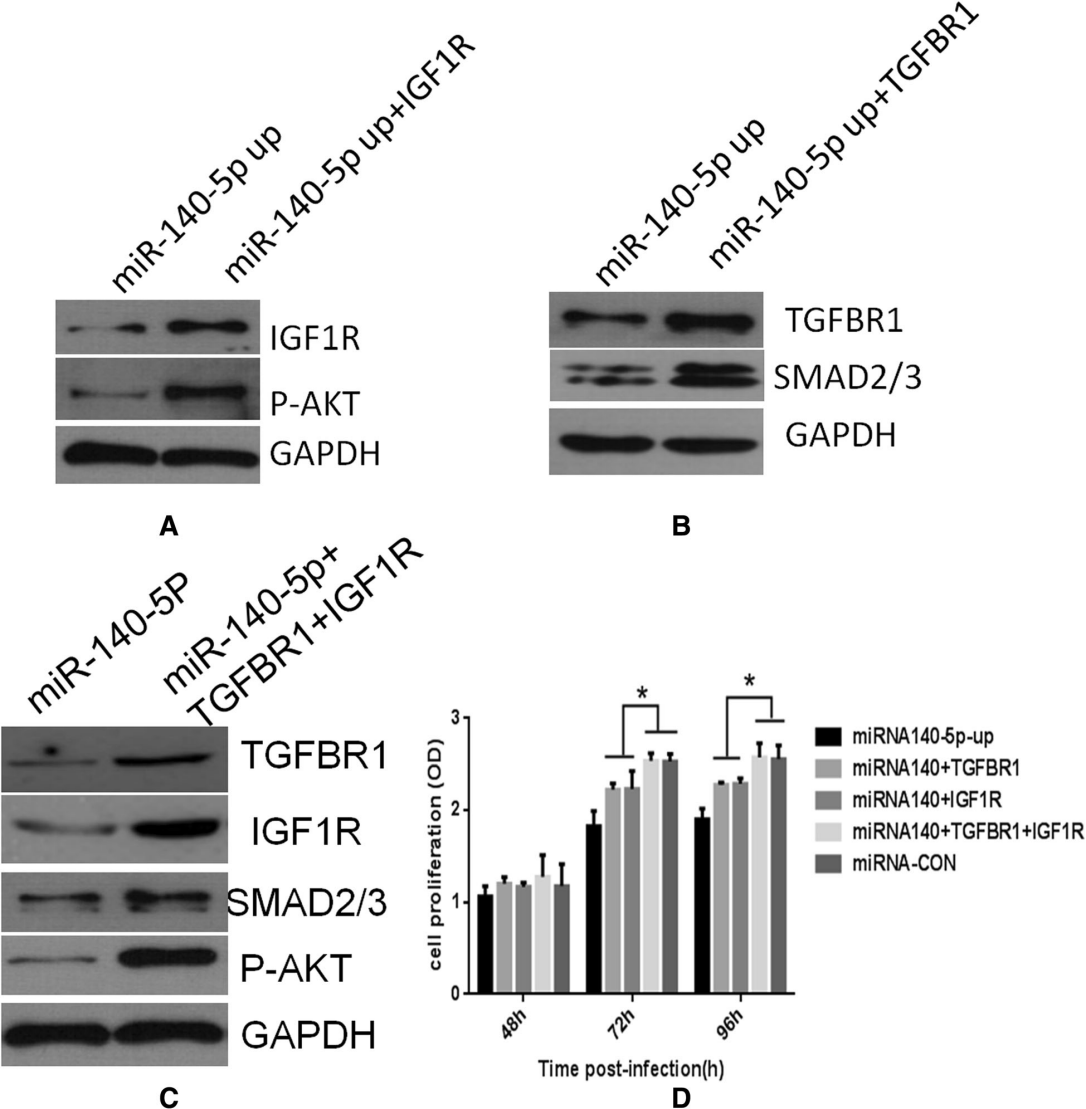
Overexpression of TGFBRI and IGF1R could abolish the inhibitory effects of microRNA-140-5p. Exogenous TGFBRI and IGF1R were introduced into microRNA-140-5p overexpressed G401 cells. Overexpression of IGF1R could rescue activation of AKT and overexpression of TGFBRI could rescue SMAD2/3 expression (**a**, **b** and **c**). Overexpression of TGFBRI and IGF1R could rescue cell proliferation rate inhibited by miR-140-5p in G401 cells. ^*^P < 0.05 (**d**)

Having shown that TGFBR1 and IGF-1R are direct targets of miR-140-5p and that they participate in cell proliferation, we further tested whether ectopic expression of TGFBR1 and IGF-1R could rescue the effect caused by miR-140-5p. Ectopic expression of TGFBR1 or IGF-1R in miR-140-5p-transduced G401 cells could abrogate inhibition of SMAD2/3 or p-AKT by miR-140-5p (Fig. 5 a, b), and partially attenuate the inhibitory effects of miR-140-5p on cell proliferation (Fig. 5 d). In contrast to these observations, overexpression of both TGFBR1 and IGF-1R could rescue SMAD2/3 and p-AKT expression and cell proliferation completely (Fig. 5 c and d). In summary, we propose that miR-140-5p can regulate oncogenic receptor-related cell invasiveness and proliferation in Wilms’ tumor.

## Discussion

Nephroblastoma also called Wilms’ tumor, is one of the most common solid tumours in children. Increasing evidence has suggested that multiple signaling, epigenetic and miRNA pathways are involved in its progression [24]. Aberrant miRNA expression is closely associated with various types of cancer [25, 26], and numerous miRNAs have significant functions in cancer cell proliferation, apoptosis, migration and neoplastic transformation [8, 27, 28]. In the present study, we investigated the miRNA expression profile of nephroblastoma specimens and concluded that miR-140-5p was downregulated in the tumor site of these samples.

MiR-140-5p levels have been shown to be decreased in multiple cancer types, such as hepatocellular carcinoma [8], colorectal cancer [29] and gastric cancer [30]. MiR- 140-5p upregulation can inhibit cell proliferation. Yang et al. demonstrated that miR-140-5p levels were decreased in HCC tissues and that miR-140-5p could regulate the activity of ERK/MAPK signaling by directly targeting TGFBRI during the metastatic process of hepatocellular carcinoma [8]. Yunfeng et al. demonstrated that miR-140-5p could suppress tumor growth and metastasis of non-small cell lung cancer by targeting IGF1R [9]. However, the function of miR-140-5p in the proliferation of Wilms’ tumor has not been previously studied. In the present study, we proved that TGFBR I and IGF1R are direct target of mi-140-5p, whereas the data further indicated that overexpression of miR-140-5p could modulate their expression and the proliferation and metastasis of nephroblastoma cells.

TGF-β signaling regulates the growth, differentiation, and metastasis of various types of cells. It functions both as a tumor suppressor and tumor promoter [31, 32]. In the early stages of cancer, TGF-β serves as a tumor suppressor [31]. However, in the advanced stages of cancer, TGF-β facilitates the progression and metastasis of tumors. In the present study, we found that inhibition of TGFBRI by siRNA could suppress cell proliferation and invasion that was in accordance with previous studies that examined the function of miR-140-5p during the advanced stages of cancer.

Zhai et al. identified SMAD2 as a direct target of miR-140-5p in CRC cells [33]. The data of the present study indicated that SMAD2/3 levels were reduced in WT-CLS1 and G401 cells when high levels of miR-140-5p were present. We further demonstrated that exogenous TGFBRI expression could recover the activity of SMAD2/3 in miR-140-5p-overexpressing WT-CLS1 and G401 cells. Considering that SMAD2/3 belongs to the TGF-β signaling pathway and that it is a downstream mediator of TGFBRI [34], we reasoned that inhibition of SMAD2/3 could be attributed notably to TGFBRI reduction. This finding indicated that miR-140-5p was not a direct target in Wilms’ tumor and that its function in carcinogenesis may be disease-specific. This finding indicated that SMAD2/3 was not a direct target of miR-140-5p in Wilms’ tumor and that its function in carcinogenesis may be disease-specific.

Several studies have shown that overexpression of the IGF-1 receptor (IGF-1R) constitutes a typical hallmark of the majority of cancer types [9]. Overexpression of IGF1R was associated with poor outcome in Wilms’ tumors, whereas the inhibition of IGF1R activity could decrease cancer malignancy [35]. One of the main downstream signals of IGF1R is AKT, which acts as a key regulator in cancer progression by promoting cell growth, anti-apoptotic effects, and cell invasion [36]. We demonstrated in the present study that miR-140-5p could inhibit IGF1R in G401 and WT-CLS1 cells, and that activation of p-AKT was also significantly decreased.

In the present study, we demonstrated that IGF-1R and TGFBRI were highly expressed in Wilms’ tumor tissues and that they participated in Wilms’ tumor progression. Overexpression of miR-140-5p inhibited cell progression via the IGF-1R/AKT and TGFBRI/SMAD2/3 pathways. We further demonstrated that both exogenous IGF-1R and TGFBRI in miR-140-5p overexpression cells could recover cell proliferation to normal levels, while ectopic either could not. Our data suggested that miR-140-5p regulated Wilms’ tumor progression and TGFBRI/SMAD2/3 and IGF-1R/AKT signaling pathways participate in this process.

## Conclusion

miRNA-140-5p expression was downregulated in Wilms’ tumor tissues, and miR-140-5p could regulate Wilms’ tumor progression via the IGF-1R/ AKT and TGFBRI/SMAD2/3 pathways and that it might have tumor suppressive functions with regard to Wilms’ tumor progression and metastasis.

## Abbreviations

3′UTRs: 3′-untraslated regions
ANT: Adjacent non-cancerous tissue
IGF1R: Insulin-like growth factor 1 receptor
TGFBRI: Transforming growth factor-beta type I receptor
